# Laboratory markers to identify acute histological chorioamnionitis in febrile parturients undergoing epidural analgesia: a retrospective study

**DOI:** 10.1186/s12884-023-06026-1

**Published:** 2023-11-02

**Authors:** Chenyang Xu, Chong Fan, Jingjing Zhang, Xin Zeng, Yuru Fan, Shanwu Feng

**Affiliations:** 1 Department of Anesthesiology, Women’s Hospital of Nanjing Medical University, Nanjing Maternity and Child Health Care Hospital, Nanjing, 210001 Jiangsu China; 2 Department of Emergency, Women’s Hospital of Nanjing Medical University, Nanjing Maternity and Child Health Care Hospital, Nanjing, 210001 Jiangsu China; 3 Department of Delivery Room, Women’s Hospital of Nanjing Medical University, Nanjing Maternity and Child Health Care Hospital, Nanjing, 210001 Jiangsu China; 4 Department of Medical Research Center, Women’s Hospital of Nanjing Medical University, Nanjing Maternity and Child Health Care Hospital, Nanjing, 210001 Jiangsu China

**Keywords:** Biomarkers, C-reactive protein, Epidural hyperthermia, Histological chorioamnionitis, Monocyte-lymphocyte ratio, Neutrophil-lymphocyte ratio, Placental pathology, Pathological staging

## Abstract

**Background:**

This study aimed to investigate the effect of the pathological staging of acute histological chorioamnionitis (HCA) on laboratory indicators and to conduct further studies to reassess the threshold values used by clinicians to identify acute HCA in febrile parturients undergoing epidural analgesia.

**Methods:**

A retrospective study of febrile mothers receiving epidural analgesia at Nanjing Maternal and Child Health Care Hospital from January 1, 2018 to December 31, 2018. The participants were grouped by the progression of acute HCA, and the laboratory parameters were compared between groups. The ability of C-reactive protein (CRP), neutrophil-lymphocyte ratio (NLR), monocyte-lymphocyte ratio (MLR), and monocyte-leukocyte ratio (M%), alone or in combination, to identify acute HCA in febrile parturients undergoing epidural analgesia was assessed using logistic regression and ROC curves.

**Results:**

The area under the curve (AUC) of the best logistic regression model predicting HCA climbed to 0.706 (CRP + MLR). Maternal CRP, NLR, and MLR significantly and progressively increased with the progression of acute HCA (*p* < 0.0001). Based on the ROC curves, the following thresholds were selected to define increased laboratory indicators for identifying acute HCA: CRP ≥ 6.90 mg/L, NLR ≥ 11.93, and MLR ≥ 0.57. In addition, the AUC of the best logistic regression model predicting HCA ≥ stage 2 was 0.710, so these inflammatory markers were more precise in predicting HCA ≥ stage 2.

**Conclusion:**

Increased CRP (≥ 6.90 mg/L), NLR (≥ 11.93), and MLR (≥ 0.57) may help clinicians to identify early potential acute HCA in febrile parturients receiving epidural analgesia and to monitor progression to optimize clinical treatment options.

**Trial registration:**

The study was registered in the Chinese Clinical Trial Registry on November 24, 2021 (http://www.chictr.org.cn, ChiCTR2100053554).

**Supplementary Information:**

The online version contains supplementary material available at 10.1186/s12884-023-06026-1.

## Introduction

The term “chorioamnionitis” literally refers to inflammation of the chorionic and amniotic layers of the fetal membranes, and its use conveys the etiology of microbial infection when this may not always be the case [1]. The definition of chorioamnionitis changes depending on the key diagnostic criteria, which can be clinical (typical signs and symptoms), microbiological (culture of amniotic fluid or chorioamnion), or histopathological (microscopic evidence of infection or inflammation on the placenta or chorioamnion) [2]. Histopathology suggests that neutrophilic inflammation of the placental tissue caused by an ascending bacterial infection is the gold standard for the diagnosis of acute chorioamnionitis [3]. Neutrophils of maternal origin spread from the decidua, invade the chorion and amnion, and enter the amniotic cavity [4, 5]. Fetal infection is the most advanced and dangerous stage of ascending intrauterine infection [6]. Therefore, early diagnosis of acute histological chorioamnionitis (HCA) is important for perinatal outcomes.

The most important clinical symptom of chorioamnionitis is maternal fever [7], and the biological fluids (e.g., amniotic fluid, vaginal secretions, serum or plasma, or both, and urine) of febrile pregnant women have been explored [1]. C-reactive protein (CRP) [8, 9], white blood cell (WBC) [10], neutrophil-lymphocyte ratio (NLR) [11], procalcitonin (PCT) [12], interleukin-6 (IL-6) [13], tumor necrosis factor-α (TNF-α) [14], and other pyrogenic cytokines have been assessed, and many of these inflammation-related cytokines have been linked to HCA. Studies of cytokines have improved the understanding of the mechanisms of maternal and fetal inflammation, but few biomarkers have shown clinical utility. Approximately 20% of delivery women who receive epidural analgesia have epidural hyperthermia [15], which is a non-infectious fever that can limit the diagnostic performance of fever for chorioamnionitis [16].

Although non-invasive laboratory markers have been thoroughly explored as a prenatal diagnosis of febrile maternal HCA, the link with pathological staging has received little attention. Therefore, this study aimed to investigate the effect of the pathological staging of acute HCA on laboratory indicators and to conduct further studies to reassess the threshold values used by clinicians to identify acute HCA in febrile parturients undergoing epidural analgesia.

## Materials and methods

**Fig. 1:**
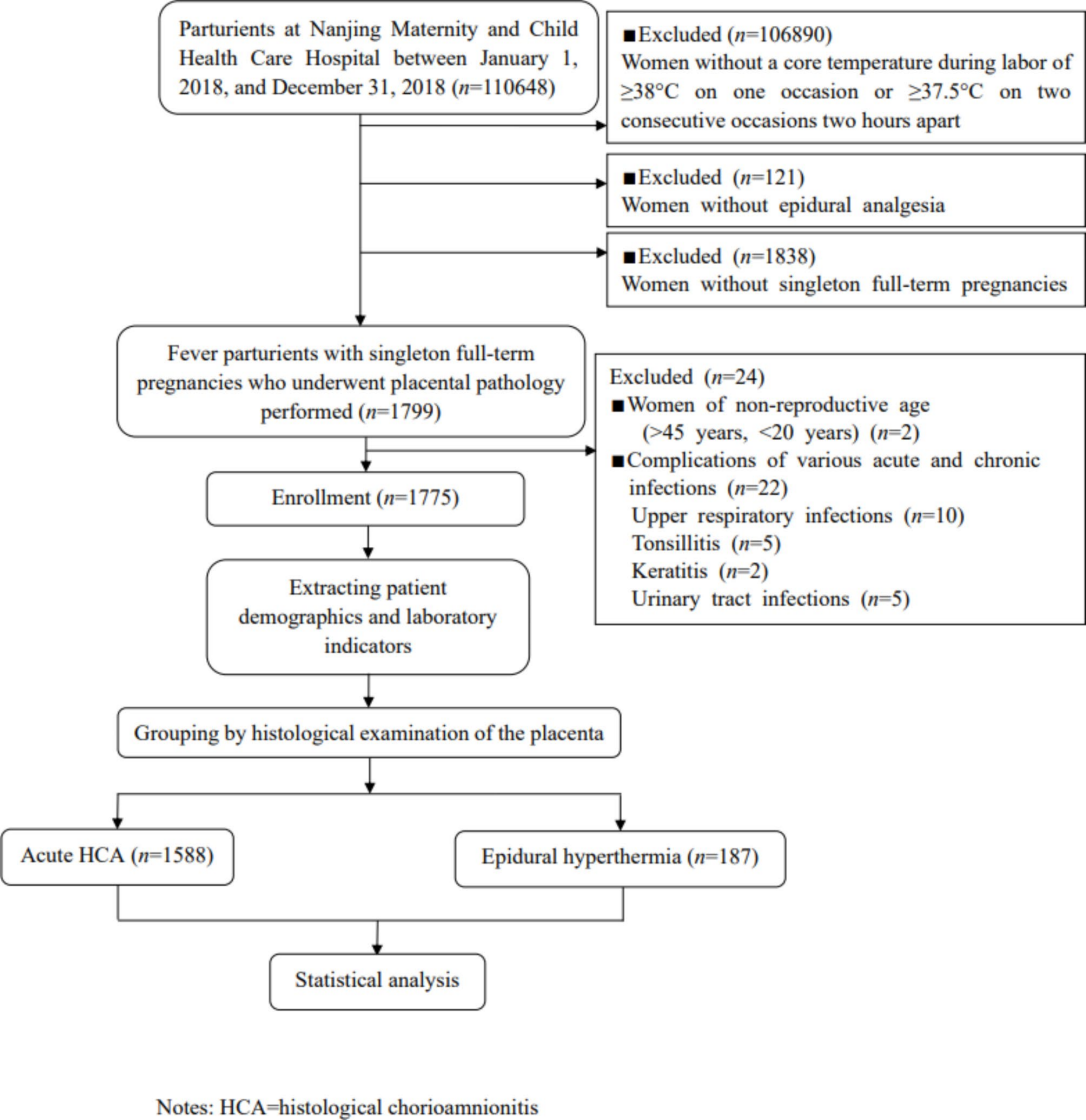
Flow diagram outlining the study procedure

This was a population-based diagnostic accuracy study including febrile parturients who received epidural analgesia at Nanjing Maternity and Child Health Care Hospital between January 1, 2018, and December 31, 2018. Because the retrospective clinical investigation caused no damage to the patients involved, the Medical Ethics Committee of Nanjing Maternity and Child Health Care Hospital agreed to waive informed consent (Approval Number: 2020 KY-035). The flow chart of the study procedure is presented in Fig. 1. All included febrile mothers were singleton term pregnancies (37 weeks ≤ gestational age ≤ 42 weeks) with placental pathology performed. Exclusion criteria were: (1) women of non-reproductive age, and (2) complications of various acute and chronic infections.

### Data extraction

Patient demographics and laboratory indicators were extracted from electronic medical record system. Demographic data include maternal age, gestational week, gravidity, parity, meconium-stained amniotic fluid (MSAF) [17], oxytocic protocol, sex and weight of the newborn, degree of perineal laceration, labor time, and amount of bleeding. Laboratory data collected included CRP, WBC, neutrophil (N), monocyte (M), lymphocyte (L), red blood cell (RBC), and platelet (PLT) counts, along with hemoglobin (Hb) levels. N%, M% and L% were calculated as the ratio of neutrophil count to white blood cell count, monocyte count to white blood cell count and lymphocyte count to white blood cell count, respectively. NLR and monocyte-lymphocyte ratio (MLR) were defined as the ratio of neutrophil count to lymphocyte count and monocyte count to lymphocyte count, respectively. Routine maternal care includes temperature monitoring, especially for women receiving epidural analgesia. Once a maternal temperature of ≥ 37.5 °C was detected, a maternal blood sample was collected by anterior elbow venipuncture and sent for testing, and laboratory indications of the appeal were obtained approximately half an hour later. In addition, if a patient receives two or more blood tests after a fever, we use the results of the first blood test to assure consistency and timeliness for all patients.

### Diagnostic criteria for acute HCA

**Fig. 2:**
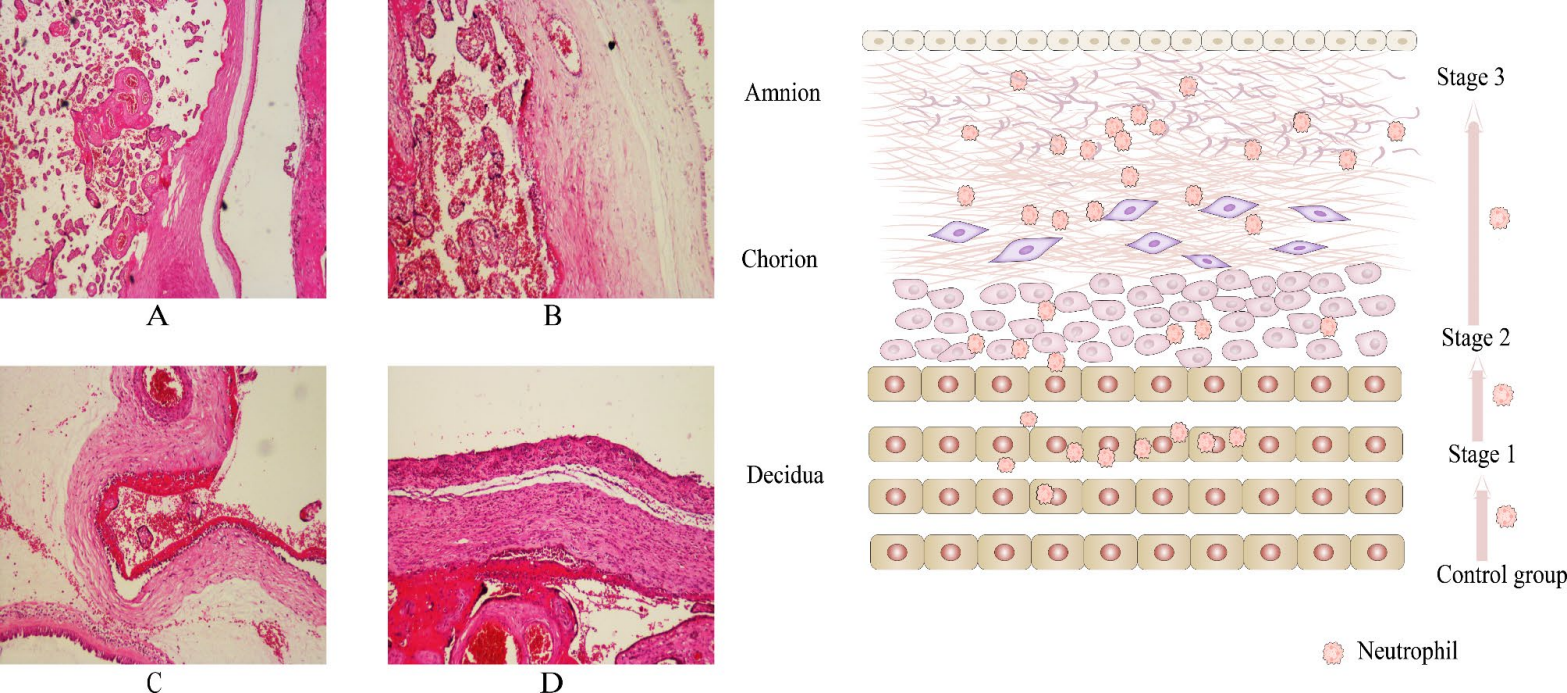
Histopathology of the progression of acute histological chorioamnionitis (HCA). Hematoxylin and eosin-stained placental tissue sections showed as follows: (**A**) Control group; (**B**) Stage 1; (**C**) Stage 2; (**D**) Stage 3. Diagnosis of acute HCA according to our hospital criteria: The number of neutrophils per field was ≥ 5 at per high-power field (HPF) (magnification, ×200). Some neutrophils are found in the decidua, the membrane trophoblast of the chorion, the connective tissue of the chorion and the amnion (the insets of panels, magnification, ×400), with the arrow indicating the outward to inward infiltration of neutrophils into the placenta (A-D)

Histologic examination of the placenta is performed in febrile pregnant women as part of routine clinical care. Collected placentas were fixed in 10% neutral buffered formalin, paraffin embedded, sectioned, and stained with hematoxylin and eosin (H&E) for histological examination. Acute HCA is defined as placental tissue (amnion, chorion, decidua, umbilical cord, or chorionic plate) with mild to severe acute inflammatory alterations. The progression of acute HCA according to the Blanc’s classification [10, 18, 19], based on the outward to inward migration of neutrophils in the placenta, is classified as follows (Fig. 2) : (1) Control group, non-inflamed placenta; (2) Stage 1, neutrophils confined to the cellulose under the chorionic plate or within the decidual layer of the fetal membrane; (3) Stage 2, neutrophils infiltrating the chorionic membrane without reaching the amnion; (4) Stage 3, neutrophils extensively infiltrating the decidual and chorionic membrane and reaching the amnion.

### Diagnostic criteria for epidural hyperthermia

Any elevated maternal temperature during labor is referred to as intrapartum hyperthermia [20]. Because fever suggests an inflammatory process, which has not been proved, hyperthermia is the recommended word [20]. A commonly used definition of intrapartum hyperthermia is: ‘a core temperature during labor of ≥ 38°C on one occasion or ≥ 37.5°C on two consecutive occasions two hours apart’ [20, 21]. Epidural hyperthermia and intrapartum infection are the two main etiologies [20]. Epidural hyperthermia, also known as epidural-related maternal fever, refers to the placental pathological examination of pregnant women with fever who use epidural labor analgesia, and no basis for infection is found [21]. At present, there is no treatment for epidural hyperthermia, and it is not possible to distinguish epidural hyperthermia from other causes of parturient hyperthermia [20]. Intrapartum infection has potentially devastating consequences for the mother and newborn, so management of intrapartum hyperthermia should include paracetamol, blood cultures, antibiotic administration, and supportive measures [20, 22].

### Grouped by disease type

20% of febrile parturients may have both epidural hyperthermia and intrapartum infection, suggesting that the two conditions are not entirely independent [20]. Therefore, patients with only epidural hyperthermia were utilized in this study as a control group without chorioamnionitis, and those who had it were categorized as having stage 1, stage 2, or stage 3 chorioamnionitis using Blanc’s criteria [10, 18, 19].

### Statistical analysis

Data were analyzed using SPSS software (version 25.0, SPSS Inc., Chicago, IL, USA), and plotted using GraphPad Prism 9.0.0 software (GraphPad Software, CA, USA), Origin Pro 2021 (Origin Lab Co., Northampton, MA), and Adobe Illustrator (Adobe Systems, Waltham, Mass). All continuous variables were tested for normality using the Shapiro-Wilk Test. Continuous data are expressed as mean ± standard deviation, and categorical data are expressed as number (percentages). Clinical characteristics of different groups were compared by the chi-square test for categorical variables and ANOVA for continuous variables, and those variables that were statistically different were screened for the next step of the analysis. A multivariate logistic regression was used to develop a predictive model to analyze the predictive factors associated with HCA. The predictive values of single laboratory indicators and the multivariate model were evaluated using receiver operating characteristic (ROC) analysis and the area under the curve (AUC). The variables needed to make up the best regression model were screened based on the AUC of the logistic regression model. ROC curves were created to identify the optimal dichotomized cutoff scores of independent predictors to distinguish between patients with and without HCA. Utilizing the cutoff scores, we compared the frequency of increased predictors according to the progression of acute-HCA with Pearson’s chi-square test on the one hand, and assessed the predictive performances by calculating sensitivity, specificity, positive and negative predictive values, and positive and negative likelihood ratios on the other hand. All statistical tests were two-sided, and statistical significance was defined as *P <* 0.05.

## Results

A total of 1775 patients were included in this study, of which 187 were epidural hyperthermia (control group) and 1588 were HCA. Of the 1588 patients with acute HCA, 231 had stage 1, 175 had stage 2, and 1182 had stage 3. There were no significant differences in the maternal age, gestational week, gravidity, parity, turbidity of amniotic fluid, oxytocic manner, sex and weight of the newborn, degree of perineal laceration, and amount of bleeding between the different stages of acute HCA and the control group. Only labor duration was substantially extended with the progression of acute HCA among the clinical features (Supplementary Table 1). In the laboratory indicators, compared with the control group, the acute HCA group had significantly higher levels of CRP, WBC, N, N%, M, M%, NLR, and MLR, and significantly lower levels of L and L%, but no significant differences in RBC, Hb, or PLT levels (Table 1). Labor had not yet ended at the time of maternal fever in general, so we removed the labor duration from the regression analysis.

**Table 1:** Laboratory indicators according to the progression of acute HCA

Laboratory indicators	Control group (*n* = 187)	Stage 1 (*n* = 231)	Stage 2 (*n* = 175)	Stage 3 (*n* = 1182)	*P*
CRP (mg/L)	8.49 ± 6.32	13.51 ± 11.35	14.21 ± 12.90	14.81 ± 13.91	< 0.0001
WBC (10^9^/L)	14.18 ± 3.12	14.56 ± 2.94	14.80 ± 3.31	14.97 ± 3.06	0.015
N (10^9^/L)	12.30 ± 3.02	12.70 ± 2.79	12.98 ± 3.12	13.09 ± 2.89	0.005
N%	86.29 ± 4.09	86.89 ± 3.90	87.46 ± 3.11	87.20 ± 3.43	0.025
M (10^9^/L)	0.64 ± 0.23	0.73 ± 0.26	0.70 ± 0.24	0.75 ± 0.26	< 0.0001
M%	4.60 ± 1.43	5.00 ± 1.45	4.80 ± 1.31	5.04 ± 1.44	< 0.0001
L (10^9^/L)	1.23 ± 0.40	1.12 ± 0.36	1.10 ± 0.37	1.11 ± 0.36	0.002
L%	8.98 ± 3.34	8.00 ± 3.06	7.59 ± 2.47	7.62 ± 2.77	< 0.0001
NLR	11.21 ± 5.08	12.61 ± 5.42	13.08 ± 5.40	13.31 ± 6.47	< 0.0001
MLR	0.56 ± 0.21	0.69 ± 0.27	0.69 ± 0.30	0.74 ± 0.35	< 0.0001
RBC (10^12^/L)	4.04 ± 0.37	4.09 ± 0.41	4.1 ± 0.40	4.07 ± 0.40	0.345
PLT (10^9^/L)	178.29 ± 49.41	185.43 ± 53.91	187.09 ± 51.07	185.61 ± 53.05	0.252
Hb (g/L)	119.99 ± 14.53	122.61 ± 12.10	122.52 ± 11.92	121.01 ± 12.16	0.106

HCA, histological chorioamnionitis; CRP, C-reactive protein; WBC, white blood cell; N, neutrophil; N%, neutrophil-white blood cell ratio; M, monocyte; M%, monocyte-white blood cell ratio; L, lymphocyte; L%, lymphocyte-white blood cell ratio; NLR, neutrophil-lymphocyte ratio; MLR, monocyte-lymphocyte ratio; RBC, red blood cell; PLT, platelet; Hb, hemoglobin

**Fig. 3:**
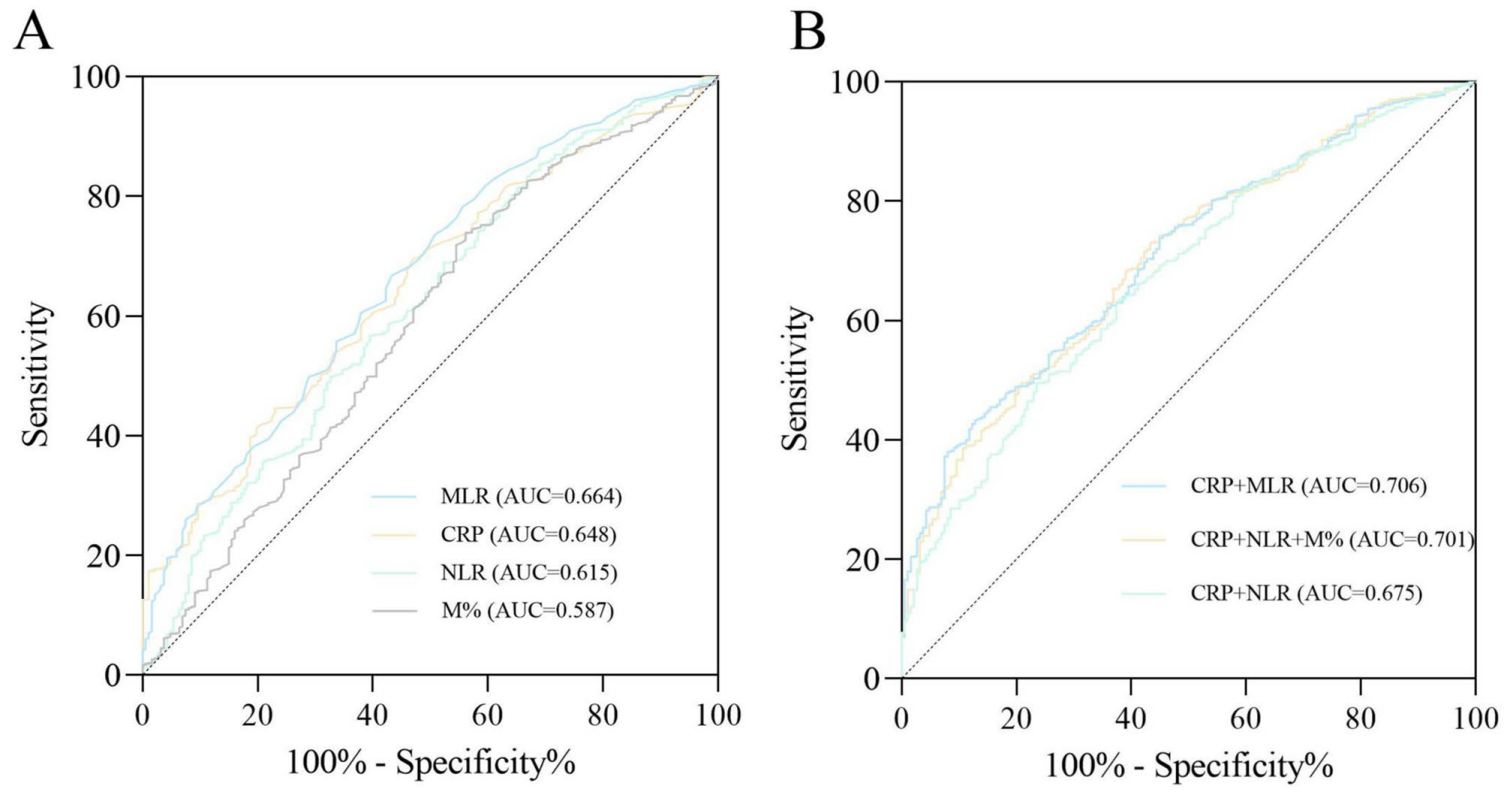
(**A**) Receiver operating characteristic (ROC) curves of single laboratory indicators for the diagnosis of acute HCA. Monocyte-lymphocyte ratio (MLR) has a higher predictive value for HCA than C-reactive protein (CRP), neutrophil to lymphocyte ratio (NLR) and monocyte-leukocyte ratio (M%). (**B**) ROC curves of combined multiple laboratory indicators for the diagnosis of acute HCA. The area under the curve (AUC) of the best logistic regression model climbed to 0.706 (CRP + MLR, 95% confidence interval, 0.671–0.742)

The logistic regression models for predicting HCA with laboratory parameters were as follows: Logit (*P*)_1_ = 0.108 + 0.058×CRP + 2.219×MLR (CRP, odds ratio, 1.060, 95% confidence interval, 1.037–1.083; MLR, odds ratio, 9.195, 95% confidence interval, 4.434–19.071), and Logit (*P*)_2_ = -0.980 + 0.059×CRP + 0.094×NLR + 0.278×M% (CRP, odds ratio, 1.061, 95% confidence interval, 1.038–1.085; NLR, odds ratio, 1.099, 95% confidence interval, 1.057–1.142; M%, odds ratio, 1.321, 95% confidence interval, 1.171–1.489). Maternal MLR, CRP, NLR, and M% are important risk factors for acute HCA, and ROC curves were constructed to assess their ability to identify acute HCA. Compared to independent factors (MLR, AUC = 0.664, 95% confidence interval, 0.624–0.705; CRP, AUC = 0.648, 95% confidence interval, 0.610–0.687; NLR, AUC = 0.615; 95% confidence interval, 0.571–0.658; M%, AUC = 0.587, 95% confidence interval, 0.542–0.632; Fig. 3A), the AUC was greater using logistic regression models that included multiple laboratory indicators (CRP + MLR, AUC = 0.706, 95% confidence interval, 0.671–0.742; CRP + NLR + M%, AUC = 0.701, 95% confidence interval, 0.665–0.738, CRP + NLR, AUC = 0.675, 95% confidence interval, 0.636–0.714; Fig. 3B).

**Fig. 4:**
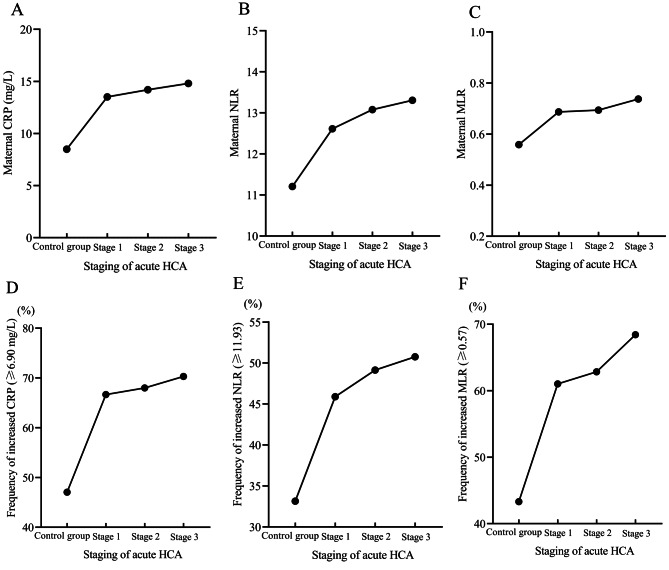
Maternal CRPs, NLRs, and MLRs increased with the progression of HCA. (**A**) Maternal CRPs (control group vs. stage 1 vs. stage 2 vs. stage 3, mean ± standard deviation, 8.49 ± 6.32 vs. 13.51 ± 11.35 vs. 14.21 ± 12.90 vs. 14.81 ± 13.91, *p* < 0.0001) significantly and progressively increased with the progression of acute HCA. (**B**) NLRs (11.21 ± 5.08 vs. 12.61 ± 5.42 vs. 13.08 ± 5.40 vs. 13.31 ± 6.47, *p* < 0.0001) significantly and progressively increased with the progression of acute HCA. (**C**) MLRs (0.56 ± 0.21 vs. 0.69 ± 0.27 vs. 0.69 ± 0.30 vs. 0.74 ± 0.35, *p* < 0.0001) significantly and progressively increased with the progression of acute HCA. (**D**) Frequency of increased CRP (≥ 6.90 mg/L) (47.06% vs. 66.67% vs. 68.00% vs. 70.30%, *p* < 0.0001) significantly and progressively increased with the progression of acute HCA. (**E**) Frequency of increased NLR (≥ 11.93) (33.16% vs. 45.89% vs. 49.14% vs. 50.76%, *p* < 0.0001) significantly and progressively increased with the progression of acute HCA. (**F**) Frequency of increased MLR (≥ 0.57) (43.32% vs. 61.04% vs. 62.86% vs. 68.44%, *p* < 0.0001) significantly and progressively increased with the progression of acute HCA

Based on the ROC curves, the following thresholds were selected to define increased laboratory indicators for identifying acute HCA: CRP ≥ 6.90 mg/L, NLR ≥ 11.93, and MLR ≥ 0.57. Similar to the increasing trend of maternal CRPs (Fig. 4A), NLRs (Fig. 4B), and MLRs (Fig. 4C) in Table 1, we found that the frequencies of maternal increased CRP (Fig. 4D), NLR (Fig. 4E), and MLR (Fig. 4F) also increased gradually with the progression of acute HCA. Finally, we evaluated the threshold used to identify HCA patients by computing the sensitivity, specificity, positive and negative predictive values, and positive and negative likelihood ratio (Table 2).

**Table 2:** Diagnostic indices, predictive values, and likelihood ratios of laboratory indicators cutoffs for the identification of acute HCA

Explanatory Variables	Sensitivity	Specificity	Positive predictive value	Negative predictive value	Positive LR	Negative LR
CRP ≥ 6.9 mg/L	0.695	0.529	92.62%	16.98%	1.476	0.577
	(1104/1588)	(99/187)	(1104/1192)	(99/583)		
NLR ≥ 11.93	0.499	0.668	92.74%	13.57%	1.503	0.582
	(792/1588)	(125/187)	(792/854)	(125/921)		
MLR ≥ 0.57	0.668	0.567	53.51%	16.72%	1.543	0.586
	(1060/1588)	(106/187)	(1060/1981)	(106/634)		

HCA, histological chorioamnionitis; CRP, C-reactive protein; NLR, neutrophil-lymphocyte ratio; MLR, monocyte-lymphocyte ratio; LR, likelihood ratio

In addition, stage 2 HCA had higher pathological and clinical significance than stage 1 HCA, so we reclassified two groups (control group vs. stage 2 and 3 HCA) to perform binary logistic regression analysis. The best logistic regression models for predicting HCA ≥ stage 2 with laboratory parameters were as follows: Logit (*P*) = -0.096 + 0.059×CRP + 2.269×MLR (CRP, odds ratio, 1.061, 95% confidence interval, 1.038–1.084; MLR, odds ratio, 9.672, 95% confidence interval, 4.633–20.191). The best logistic regression model had an AUC of 0.710 (95% confidence interval, 0.675–0.746), so these inflammatory markers were more precise in predicting HCA ≥ stage 2.

Based on the ROC curves, the following thresholds were selected to define increased laboratory indicators for identifying acute HCA ≥ stage 2: CRP ≥ 6.90 mg/L (sensitivity, 0.700; specificity, 0.529), NLR ≥ 11.93 (sensitivity, 0.506; specificity, 0.674), and MLR ≥ 0.57 (sensitivity, 0.675; specificity, 0.572).

## Discussion

### Principal findings

The logistic regression model for predicting acute HCA with multiple laboratory indicators (CRP + MLR or CRP + NLR + M%) has a higher AUC than any single laboratory indicator. Based on the staging of placental pathology, this study found that CRP, NLR, and MLR increased gradually with the progression of acute HCA. These inflammatory markers were more accurate in predicting HCA ≥ stage 2 than in predicting HCA.

### Results in the context of what is known

Bacterial staining and biochemical analysis of amniotic fluid and histopathological examination of the placenta are important methods for the diagnosis of placental inflammation [23]. The most accurate test is a culture of the amniotic fluid, but its usefulness is restricted because it can take up to three days to receive the results [24]. Additionally, amniocentesis is not carried out in the majority of situations, which take place during labor, due to the intrusive nature of the technique [24]. The lag in placental sampling also affects the uncertainty of treatment options. Previous mechanistic investigations of acute HCA have demonstrated that microbial invasion of the placenta generates a substantial local inflammatory response, as well as a significant rise in the levels of pro-inflammatory cytokines [3]. Our study showed the significant elevation of pro-inflammatory cytokines such as CRP, NLR, MLR, and M% in the blood of patients with chorioamnionitis compared to patients with epidural hyperthermia. The widespread infiltration of neutrophils into chorioamniotic membranes is caused by a gradient of chemokine concentrations formed across the chorioamniotic membranes and the decidua [3]. Our observations support the appeal view by analyzing the inflammatory infiltration of the placenta from the outside to the inside in terms of concentration gradients of CRP, NLR, and MLR.

Although epidural analgesia does not raise the risk of intrapartum infection [25], it is difficult to distinguish between infectious fever induced by chorioamnionitis and hyperthermia generated by the epidural itself [20]. Epidural hyperthermia and intrapartum infection (chorioamnionitis) may not be completely independent, and some patients may have both epidural hyperthermia and intrapartum infection [20]. There is substantial evidence for the role of maternal inflammation in the development of epidural hyperthermia [25]. Previous studies exploring the role of noninfectious inflammatory factors in the development of epidural hyperthermia have found that, on the one hand, febrile women have higher IL-6 levels than nonfebrile mothers [25], and on the other hand, both febrile and nonfebrile women undergoing epidural anesthesia have higher levels of IL-6 than those who did not receive analgesia [26]. There is no consensus on the role of inflammatory markers in the identification of chorioamnionitis in women because of the low sensitivity and specificity of laboratory indicators of maternal blood when applied alone in previous studies [10, 12, 13, 27]. Higher levels of cytokines in maternal serum indicate that the maternal compartment is the primary inflammatory source [28], and maternally derived inflammatory factors may directly induce fetal injury [29]. The inflammatory markers explored in this study were designed precisely to capitalize on differences in levels of inflammatory factors to identify the occurrence of acute HCA in febrile women receiving epidural analgesia. According to the ROC curve analysis, MLR had a higher predictive value for HCA than CRP or NLR, but the composite models had greater predictive values (AUC = 0.706). These inflammatory markers were more precise in predicting HCA ≥ stage 2 (AUC = 0.710). The thresholds of inflammatory markers for identifying HCA and HCA ≥ stage 2 were the same (CRP ≥ 6.90 mg/L, NLR ≥ 11.93, and MLR ≥ 0.57), with similar sensitivity and specificity. So clinically, you can refer to the threshold of these inflammatory indicators, combined with clinical symptoms, to be timely and effective cooling treatment.

In the early stages of chorioamnionitis, infection and inflammation are limited to the chorion and amnion membranes, and IL-6 is released into the maternal blood, leading the mother to produce CRP [8]. As a result, CRP can be used as a screening diagnostic for chorioamnionitis since it is an indirect biomarker of IL-6 secretion. The NLR has been proposed as an additional marker of infection since the normal immunological response of circulating leukocytes to systemic inflammation is accompanied by increased neutrophils and reduced lymphocytes [11]. Previous research has demonstrated that combining CRP and NLR can increase the prognosis accuracy of chorioamnionitis patients [30]. Although we evaluated the diagnostic validity of M% as an additional laboratory indicator of CRP and NLR to predict the occurrence of acute HCA, the ability of M% to independently diagnose acute HCA was weak. In addition, MLR has been proposed as a new biomarker of inflammation and infectiousness [31]. Consistent with this, the first logistic regression model of this study proposed that MLR is a predictive index of acute HCA.

### Clinical implications

The main clinical signs associated with infected chorioamnionitis include uterine tenderness, maternal tachycardia (≥ 100 beats/min), fetal tachycardia (≥ 160 beats/min), maternal leukocytosis (≥ 15 × 10^9^/L), and foul-smelling amniotic fluid, in addition to maternal fever [1, 32]. These clinical criteria are neither sensitive nor specific and may lead to misdiagnosis of HCA [16]. Therefore, some obstetricians even only consider the fever symptoms as the clinical index of antibiotic treatment. The American College of Obstetricians and Gynecologists (ACOG) guidelines define peripartum fever as a maternal temperature ≥ 38 °C (100.4 °F) [33], while the National Institutes of Health (NIH) has organized a workshop of experts to propose stricter definitions. The workshop classified maternal body temperature ≥ 39 °C (102.2 °F) or two body temperature ≥ 38 °C (100.4 °F) as “isolated maternal fever” and included it as “suspected intra-amniotic infection” (i.e., Triple I) diagnostic criteria [23]. The new term “intrauterine inflammation or infection or both” or “Triple I” instead of “chorioamnionitis” is proposed to clarify that maternal fever is not synonymous with infection or inflammation [1]. However, the presence of epidural hyperthermia in primiparous women with long labor can lead to difficulties in the diagnosis and treatment of chorioamnionitis [33].

Intrapartum fever can be classified as infectious or non-infectious depending on the etiology. Acute HCA is characterized by a diffuse infiltration of neutrophils into the chorion and amniotic membrane. Although the immunomodulatory hypothesis of epidural hyperthermia suggests that the sterile febrile response induced by epidural local anesthetics also occurs in conjunction with a proinflammatory state [20], prior studies have found that antibiotics are ineffective in preventing epidural hyperthermia [34]. Maternal antibiotic therapy has been found to increase the risk of neonatal bacteremia [35], but overuse of antibiotics following maternal fever is currently on the rise in some areas. Therefore, the prenatal biomarkers obtained in this study are important for screening acute HCA and may provide a basis for clinical decision-making.

### Research implications

We limited the study population to febrile parturients, which can guide clinical management after maternal fever compared to other previous studies [2, 16]. In this study, it was not possible to include the three laboratory indicators of CRP, NLR, and MLR in the logistic regression analysis, and a study with a larger sample may be needed to evaluate the combined diagnostic performance of the three. Considering the sensitivity and specificity of individual laboratory indicators, we recommend the inclusion of maternal CRP, NLR, and MLR as combined indicators to identify the occurrence of acute HCA in febrile parturients receiving epidural analgesia.

### Strengths and limitations

First, this study showed the progressive increase of inflammatory factors (CRP, NLR, and MLR) with the progression of acute HCA. Second, unlike previous studies, this study proposes that maternal MLR is a biomarker for the diagnosis of acute HCA. Third, this study recommends maternal CRP, NLR, and MLR as combined maternal biomarkers that can be widely used in basic medical institutions to distinguish epidural hyperthermia from acute HCA. The diagnostic criteria for chorioamnionitis varied depending on the type of classification used. Therefore, hospitals using the Blanc’s classification may be able to use the results of this study as a reference, while hospitals using other classifications cannot [36]. The inclusion of febrile maternity varies by definition. The inclusion of mothers with a temperature of 37.5 °C in fever cases is a limitation of this study.

However, since previous placental histopathology was limited to febrile parturients, only febrile parturients were included in this study. Further diagnostic accuracy studies are needed to screen for subclinical HCA in parturients without fever to verify the applicability of the results in this study. In addition, this study only included women with singleton term pregnancies, and further studies are needed to determine whether there are similar biomarkers for febrile women with early and late labor. Since we use individual blood samples during fever to calculate inflammatory factors, we were unable to reflect long-term inflammatory status. The positive and negative likelihood ratios for the three prenatal biomarker cutoffs used to identify acute HCA in this study remain low.

## Conclusions

Maternal CRP, NLR, and MLR increased significantly and progressively with the progression of acute HCA, and increased CRP (≥ 6.90 mg/L), NLR (≥ 11.93), and MLR (≥ 0.57) may be considered as biomarkers to identify the development of acute HCA in febrile parturients receiving epidural analgesia. These indicators may help clinicians identify potential acute HCA patients early and monitor disease progression to optimize clinical treatment options.

### Electronic supplementary material

Below is the link to the electronic supplementary material.

Supplementary Material 1

## Data Availability

The datasets generated during and analyzed during the current study are not publicly available due to data protection reasons, but are available from the corresponding author (Shanwu Feng, Email: shanwufeng666@163.com) on reasonable request.

## List of abbreviations

ACOG: American College of Obstetricians and Gynecologists
AUC: Area under the curve
CRP: C-reactive protein
H&E: Hematoxylin and eosin
Hb: Hemoglobin
HCA: Histological chorioamnionitis
IL-6: Interleukin-6
L: Lymphocyte
M: Monocyte
MLR: Monocyte-lymphocyte ratio
MSAF: Meconium-stained amniotic fluid
N: Neutrophil
NIH: National Institutes of Health
NLR: Neutrophil-lymphocyte ratio
PCT: Procalcitonin
PLT: Platelet
RBC: Red blood cell
ROC: Receiver operating characteristic
TNF-α: Tumor necrosis factor-α
WBC: White blood cell
