# Correction: Laboratory markers to identify acute histological chorioamnionitis in febrile parturients undergoing epidural analgesia: a retrospective study

**DOI:** 10.1186/s12884-023-06153-9

**Published:** 2023-11-24

**Authors:** Chenyang Xu, Chong Fan, Jingjing Zhang, Xin Zeng, Yuru Fan, Shanwu Feng

**Affiliations:** 1 Department of Anesthesiology, Women’s Hospital of Nanjing Medical University, Nanjing Maternity and Child Health Care Hospital, Nanjing, 210001 Jiangsu China; 2 Department of Emergency, Women’s Hospital of Nanjing Medical University, Nanjing Maternity and Child Health Care Hospital, Nanjing, 210001 Jiangsu China; 3 Department of Delivery Room, Women’s Hospital of Nanjing Medical University, Nanjing Maternity and Child Health Care Hospital, Nanjing, 210001 Jiangsu China; 4 Department of Medical Research Center, Women’s Hospital of Nanjing Medical University, Nanjing Maternity and Child Health Care Hospital, Nanjing, 210001 Jiangsu China

**Correction: BMC Pregnancy Childbirth 23, 766 (2023)**

10.1186/s12884-023-06026-1

Following publication of the original article [1], the authors reported an error in Author’s affiliations.

The author group has been updated above and the original article [1] has been corrected.
