# Supplementary material for: Laboratory markers to identify acute histological chorioamnionitis in febrile parturients undergoing epidural analgesia: a retrospective study
Source: BMC Pregnancy Childbirth. 2023 Nov 2;23:766. doi: 10.1186/s12884-023-06026-1 (PMC10621168; doi:10.1186/s12884-023-06026-1)
Supplement: Supplementary file 1 — Supplementary Material 1 [file 12884_2023_6026_MOESM1_ESM.docx]

Supplementary Table 1. Clinical characteristics according to the progression of acute HCA.

| Characteristics | Control group  (*n*=187) | Stage 1  (*n*=231) | Stage 2  (*n*=175) | Stage 3  (*n*=1182) | *P* |
| --- | --- | --- | --- | --- | --- |
| Maternal age (year) | 28.71±2.81 | 28.50±2.79 | 28.47±2.99 | 28.49±2.96 | 0.726 |
| Gestational week | 39.24±1.12 | 39.31±1.30 | 39.25±1.23 | 39.30±1.37 | 0.435 |
| Gravidity |  |  |  |  | 0.621 |
| 1 | 134 (7.5%) | 166(9.4%) | 116(6.5%) | 828(46.6%) |  |
| >1 | 53(3.0%) | 65(3.7%) | 59(3.3%) | 354(19.9%) |  |
| Parity |  |  |  |  | 0.337 |
| 1 | 177 (10.0%) | 223 (12.6%) | 162 (9.1%) | 1124 (63.3%) |  |
| >1 | 10 (0.6%) | 8 (0.5%) | 13 (0.7%) | 58 (3.3%) |  |
| MSAF* |  |  |  |  | 0.564 |
| Clean | 133 (7.5%) | 162 (9.1%) | 114 (6.4%) | 808 (45.5%) |  |
| I | 14 (0.8%) | 14 (0.8%) | 14 (0.8%) | 98 (5.5%) |  |
| II | 22 (1.2%) | 21 (1.2%) | 16 (0.9%) | 106 (6.0%) |  |
| III | 17 (1.0%) | 32 (1.8%) | 30 (1.7%) | 167 (9.4%) |  |
| Oxytocic protocol^#^ |  |  |  |  | 0.273 |
| No | 65 (3.7%) | 75 (4.2%) | 44 (2.5%) | 340 (19.2%) |  |
| Propess | 1 (0.1%) | 3 (0.2%) | 3 (0.2%) | 5 (0.3%) |  |
| Oxytocin | 118 (6.6%) | 147 (8.3%) | 124 (7.0%) | 808 (45.5%) |  |
| Multiple ways | 3 (0.2%) | 6 (0.3%) | 4 (0.2%) | 28 (1.6%) |  |
| Newborn |  |  |  |  | 0.547 |
| Female | 97 (5.5%) | 104 (5.9%) | 83 (4.7%) | 578 (32.6%) |  |
| Male | 90 (5.1%) | 127 (7.2%) | 92 (5.2%) | 604 (34.0%) |  |
| Birth weight (g) | 3380.64±357.75 | 3399.52±379.30 | 3400.06±408.24 | 3384.04±376.11 | 0.814 |
| Degree of perineal laceration |  |  |  |  | 0.169 |
| No | 2 (0.1%) | 4 (%) | 1 (%) | 18 (%) |  |
| I | 114 (6.4%) | 117 (%) | 90 (%) | 635 (%) |  |
| II | 34 (%) | 40 (%) | 45 (%) | 236 (%) |  |
| Episiotomy | 37 (%) | 70 (%) | 39 (%) | 293 (%) |  |
| Labor time (min) | 631.58±162.38^a, b^ | 651.72±177.41^c^ | 675.71±163.54 | 693.75±174.49 | <0.0001 |
| Amount of bleeding (ml) | 303.13±75.85 | 305.33±103.45 | 313.92±80.51 | 317.454±110.19 | 0.119 |

HCA, histological chorioamnionitis; ^a^ *P*<0.05 vs. stage 2, ^b^ *P*<0.05 vs. stage 3, ^c^ *P*<0.05 vs. stage 3 (one-way ANOVA with post-hoc LSD test).

* MSAF=Meconium-stained amniotic fluid. If meconium is passed into amniotic fluid during pregnancy, it stains the fluid, leading to MSAF. MSAF was further divided into three grades: grade I was thin yellow color meconium with no particulate matter, grade II was light green color with few particulate matters, and grade III was thick paste-like dark green-colored meconium with excess particulate matter. Clean means that the amniotic fluid is not contaminated.

^#^ There are four kinds of oxytocic protocols, no stands for spontaneous delivery, propess stands for the propess vaginal pessary serving as a prostaglandin E2 slow-release system, oxytocin stands for intravenous medicine, and multiple ways stands for two or more oxytocic protocols.
